# Immunization against arthropod protein impairs transmission of rickettsial pathogen from ticks to the vertebrate host

**DOI:** 10.1038/s41541-023-00678-y

**Published:** 2023-05-30

**Authors:** P. P. Mahesh, Prachi Namjoshi, Hameeda Sultana, Girish Neelakanta

**Affiliations:** Department of Biomedical and Diagnostic Sciences, College of Veterinary Medicine, University of Tennessee, Knoxville, TN USA

**Keywords:** Protein vaccines, Bacterial infection

## Abstract

Human anaplasmosis caused by *Anaplasma phagocytophilum* is one of the most common tick-borne diseases in the United States. The black-legged ticks, *Ixodes scapularis*, vector and transmit this bacterium to humans. In this study, we provide evidence that targeting *I. scapularis* membrane-bound organic anion transporting polypeptide 4056 (IsOATP4056) with an anti-vector vaccine affects transmission of *A. phagocytophilum* from ticks to the vertebrate host. *Anaplasma phagocytophilum* induces expression of IsOATP4056 in ticks and tick cells. Increased membrane localization of IsOATP4056 was evident in *A. phagocytophilum*-infected tick cells. Treatment with high dose (10 µg/ml) but not low dose (5 µg/ml) of EL-6 antibody that targets the largest extracellular loop of IsOATP4056 showed cytotoxic effects in tick cells but not in human keratinocyte cell line (HaCaT). Passive immunization, tick-mediated transmission and in vitro studies performed with mice ordered from two commercial vendors and with tick cells showed that EL-6 antibody not only impairs *A. phagocytophilum* transmission from ticks to the murine host but also aids in the reduction in the bacterial loads within engorged ticks and in tick cells by activation of arthropod Toll pathway. Furthermore, reduced molting efficiency was noted in ticks fed on EL-6 antibody-immunized mice. Collectively, these results provide a good candidate for the development of anti-tick vaccine to target the transmission of *A. phagocytophilum* and perhaps other tick-borne pathogens of medical importance.

## Introduction

Tick-borne diseases such as Lyme borreliosis, human anaplasmosis, ehrlichiosis, Rocky Mountain spotted fever, tularemia, Powassan encephalitis, tick-borne encephalitis, Kyasanur forest disease and others are important human health threats throughout the world^1–5^. The pathogens that cause these diseases are primarily transmitted by ticks via a blood meal^1,2,4,6,7^. Abiotic and biotic factors such as climate change and availability of reservoir hosts and ticks could contribute for the geographic expansion/distribution of ticks and tick-borne diseases^8–10^. Traditional methods, such as the use of acaricides, for containing tick infestations are less effective in many instances^11^. Therefore, the development of vaccines seems to be a more effective and environmentally friendly approach to prevent these diseases.

The approval of tick-borne encephalitis vaccine (TICOVAC) provides evidence that vaccines are the ideal strategies to target/prevent tick-borne diseases^12^. However, there are no authorized vaccines to treat/prevent several other tick-borne bacterial diseases^6,13,14^. A single tick species could vector and transmit several human pathogens^15–17^. Therefore, innovative strategies that directly target tick infestation and transmission of pathogens are highly desirable^6,13,18^. Several studies have highlighted that vaccination against tick-protective antigens would prevent tick feeding and/or pathogen transmission^6,13,18–24^. Therefore, studies that characterize new conserved protective antigens are important for the development of universal anti-tick vaccine to target multiple tick species. An anti-vector vaccine candidate gives hope for targeting more than one pathogen transmitted by the same vector.

Human anaplasmosis caused by the obligate intracellular bacterium, *Anaplasma phagocytophilum*, is one the most common vector-borne diseases in the United States^25,26^. Clinical manifestations includes fever, malaise, headache, arthralgia, thrombocytopenia and leukopenia^25^. This agent is mainly transmitted by a black-legged tick, *Ixodes scapularis*. These ticks have four phases in their development that includes eggs, larvae, nymphs and adult stages^10^. Infection with *A. phagocytophilum* leads to a series of events which subvert the host signaling and help the bacterium to persist in both mammals and arthropod vectors^26–34^. A previous study from our laboratory showed that *I. scapularis* Organic Anion Transporting Polypeptide (OATP) 4056 (IsOATP4056) was upregulated during *A. phagocytophilum* infection^35^. In addition, exogeneous addition of the tryptophan pathway metabolite, xanthurenic acid (XA), to ticks and tick cells resulted in increase of both the expression of *isoatp4056* and the bacterial burden^35^. The *isoatp4056* is the only OATP among nine OATPs in ticks which was upregulated in salivary glands during *A. phagocytophilum* infection^35^. RNAi-mediated knockdown of *isoatp4056* and inhibition of OATPs with an inhibitor affected bacterial replication in ticks and tick cells^35^. We also noted that tick-borne Langat virus (LGTV) modulate OATPs in ticks and inhibition of these proteins affected the viral burden in tick cells^36^. More recently, we reported that XA can help the pathogen to evade reactive oxygen species^37^. In the follow-up study, we reported that *A. phagocytophilum* downregulates microRNA133 (miR-133) that targets *isoatp4056* mRNA^38^. Overexpression of miR-133 in ticks affected *A. phagocytophilum* transmission from ticks to the vertebrate host suggesting the importance of IsOATP4056 in this bacterial transmission^38^. We also noted that the expression of *isoatp4056* was upregulated during *A. phagocytophilum* transmission from ticks to the vertebrate host. Overall, these studies provide strong evidence that targeting IsOATP4056 could be an ideal strategy to block the transmission of *A. phagocytophilum* from ticks to the vertebrate host.

OATPs are transmembrane transporting proteins that have several extracellular and intracellular loops^39–42^. These proteins aid in the transcellular movement of various hormones, anions, signaling molecules, toxins and growth factors^39–43^. Based on the bioinformatic prediction, IsOATP4056 C-terminal portion extends out of the plasma membrane^36^, which is not observed in other tick or human OATPs. In this study, we used two affinity-purified polyclonal antibodies raised against the Extracellular Loop-2 (EL-2) and the C-terminal Extracellular Loop-6 (EL-6), for conducting in vitro and in vivo experiments. This study shows that the presence of antibody against EL-6 of IsOATP4056 significantly reduces *A. phagocytophilum* burden both in tick cells and in infected ticks fed on immunized mice. In addition, we noted that passive immunization with EL-6 antibody reduced the transmission of bacteria from ticks to the murine host. We also show that the presence of EL-6 antibody activates TOLL pathway both in ticks and tick cells and aids in the reduction in bacterial loads. This study provides evidence on the vaccine efficacy of a new tick protective antigen to target the transmission of *A. phagocytophilum* and perhaps other pathogens from ticks to the vertebrate host.

## Results

### *Anaplasma phagocytophilum* infection leads to increased expression of IsOATP4056 protein in ticks and tick cells

**Fig. 1:**
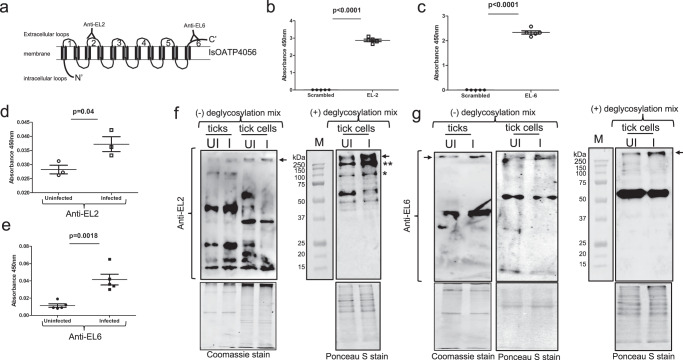
*Anaplasma phagocytophilum* upregulates IsOATP4056 in ticks and tick cells. **a** Schematic representation of IsOATP4056 organization on the tick cell plasma membrane is shown. Extracellular loops are indicated with numbers from 1-6. N- and C-terminal ends are labeled. Antibodies were generated against epitopes from EL-2 or EL-6 region. Schematics is not drawn to the scale. ELISA performed with scrambled peptide (**b** and **c**), EL-2 peptide (**b**), EL-6 peptide (**c**) probed with EL-2 or EL-6 antibody is shown. ELISA performed with whole tick lysates prepared from unfed uninfected or *A. phagocytophilum*-infected nymphal ticks probed with EL-2 (**d**) or EL-6 (**e**) antibody is shown. Each circle/square indicates data generated from one tick or tick cell sample from one culture well. *p*-value from student test is shown. Error bars indicate +/− standard error from the mean. Immunoblotting analysis with EL-2 (**f**) or EL-6 (**g**) antibody showing levels of IsOATP4056 in uninfected or *A. phagocytophilum*-infected ticks or tick cells in the presence (+) or absence (−) of deglycosylation mix is shown. Arrow indicates oligomeric form of IsOATP4056 (>250 kDa). ** indicates dimeric form of IsOATP4056 (~250 kDa) and * indicates monomeric form of IsOATp4056 (~100 kDa). M indicate marker, UI indicate uninfected, and I indicate *A. phagocytophilum*-infected ticks or tick cells.

**Fig. 2:**
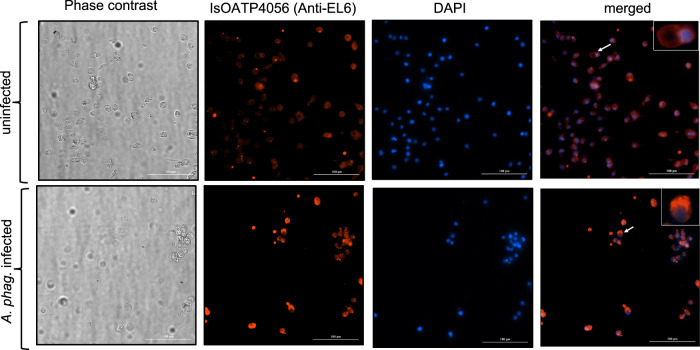
Immunofluorescence analysis showing increased expression of IsOATP4056 in *A. phagocytophilum*-infected tick cells. Phase contrast or fluorescent microscopic images showing IsOATP4056 levels using EL-6 antibody (Alexa-594, red) and nuclear staining (blue, DAPI) in uninfected or *A. phagocytophilum*-infected tick cells using EL-6 antibody. Merged images show overlay of IsOATP4056 with DAPI staining. Insert in the merged images shows the blow-out image of the cell pointed with an arrow in the full image. Scale bar indicates 100 µm.

In our previous study, we reported that *isoapt4056* transcripts were significantly upregulated upon *A. phagocytophilum* infection in ticks^35^. To analyze whether similar observation is evident at the protein level, affinity-purified polyclonal antibodies raised against IsOATP4056 Extracellular Loop-2 (EL-2) and Extracellular Loop-6 (EL-6) peptides were generated in this study (Fig. 1a). EL-6 is the largest region among six extracellular loops present in IsOATP4056 (Fig. 1a). The enzyme-linked immunosorbent assay (ELISA) showed significantly (P < 0.05) increased binding of EL-2 (Fig. 1b) and EL-6 (Fig. 1c) antibodies to the respective IsOATP4056 peptides in comparison to the binding noted with control scrambled peptide. In addition, ELISAs performed with EL-2 (Fig. 1d) and EL-6 (Fig. 1e) antibodies and whole tick, or tick cell lysates showed significantly (*P* < 0.05) increased expression of IsOATP4056 in unfed *A. phagocytophilum*-infected nymphal ticks (Fig. 1d and e) and tick cells (Supplementary Fig. 1a and b) in comparison to the levels noted in uninfected controls. Furthermore, immunoblotting analyses with EL2 or EL6 antibodies in the presence (+) or absence (−) of deglycosylation mix revealed an intense band above 250 kDa in samples generated from *A. phagocytophilum*-infected whole ticks or tick cell lysates compared to the levels noted in uninfected controls in both conditions (Fig. 1f and g). However, we noted that upon deglycosylation followed by immunoblotting with EL2 antibody but not with EL6 antibody, intense bands at ~ 250 kDa and ~100 kDA were also evident in samples generated from *A. phagocytophilum*-infected tick cells compared to the levels noted in uninfected controls (Fig. 1f and g). A two-three fold increased levels of >250 kDa band (in all conditions) and ~250 kDa and ~100 kDa bands (in deglycosylation condition followed by probing with EL-2 antibody) was evident in lysates prepared from *A. phagocytophilum*-infected tick cells when compared to the levels noted in lysates prepared from uninfected controls (Fig. 1f and g). In addition, immunofluorescence analysis performed with EL-6 antibody showed increased IsOATP4056 staining in *A. phagocytophilum*-infected tick cells compared to the staining noted in uninfected control (Fig. 2). Furthermore, increased levels of IsOATP4056 were noted to be localized on the plasma membrane of *A. phagocytophilum*-infected tick cells when compared to the levels noted on the plasma membranes of uninfected control (Fig. 2). Collectively, these results show that *A. phagocytophilum* upregulates IsOATP4056 in ticks and tick cells and enhances the localization of this protein on the plasma membrane of tick cells.

### Treatment of tick cells with EL-6 antibody reduces the bacterial burden and upregulates arthropod TOLL pathway

**Fig. 3:**
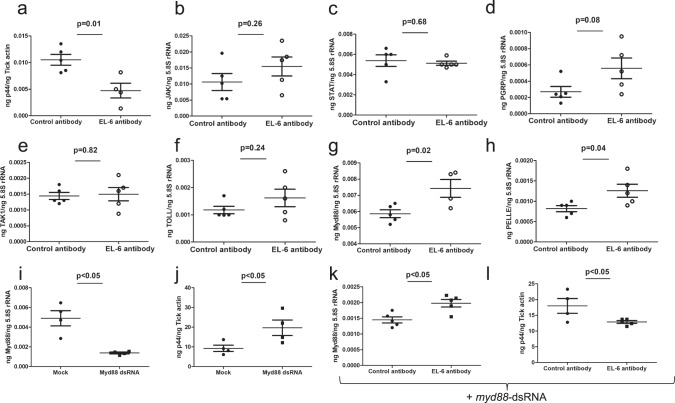
EL-6 antibody-treatment reduces bacterial burden and upregulates TOLL pathway in tick cells. QRT-PCR analysis showing bacterial burden, analyzed by *p44* levels (**a**), and *jak* (**b**), *stat* (**c**), *pgrp* (**d**), *tak1* (**e**), *toll* (**f**), *myd88* (**g**) or *pelle* (**h**) expression in control or EL-6 antibody-treated *A. phagocytophilum*-infected tick cells. Control and EL-6 antibodies were used at a concentration of 5 µg/ml. QRT-PCR analysis showing levels of *myd88* expression (**i**) or bacterial burden (**j**) in mock- or *myd88*-dsRNA-treated *A. phagocytophilum*-infected tick cells. *myd88* expression (**k**) or bacteria burden (**l**) in control- or EL-6-antibody-treated *myd88*-dsRNA-contained *A. phagocytophilum*-infected tick cells is shown. *Anaplasma phagocytophilum* p44 levels were normalized to tick actin levels and mRNA levels of tick innate immune genes were normalized to tick 5.8 S rRNA levels. *p*-value from student test is shown. Error bars indicate +/− standard error of the mean. Each circle represents data obtained from the sample collected from one well of the tick cells culture plate.

Our previous study showed that RNAi-mediated knockdown of *isoatp4056* expression resulted in reduced bacterial burden in both ticks and tick cells^35^. We now tested whether antibody-mediated blocking of IsOATP4056 has similar effect on bacterial burden in tick cells (Fig. 3 and Supplementary Fig. 2). Tick cells were treated with 5 µg/ml of EL-2 (Supplementary Fig. 2) or EL-6 (Fig. 3) antibody one day prior to infection with *A. phagocytophilum*. Bacterial burden was monitored at 24 h postinfection. QRT-PCR analysis showed that *A. phagocytophilum* burden was significantly reduced upon treatment of tick cells with EL-6 antibody compared to the burden noted in tick cells treated with control antibody (Fig. 3a). However, no significant difference in the bacterial burden was noted between EL-2 antibody-treated and control-antibody-treated tick cells (Supplementary Fig. 2). We recently showed that *A. phagocytophilum* modulates circadian CLOCK-mediated regulation of arthropod innate immune genes during its transmission from ticks^44^. We therefore reasoned whether the observation of reduced bacterial burden upon EL-6 antibody treatment is related to the activation of tick immune genes. QRT-PCR analysis revealed that arthropod Toll pathway genes, *myd88* and *pelle* were significantly (P < 0.05) upregulated in EL6-antibody-treated *A. phagocytophilum*-infected tick cells in comparison to the levels noted in control-antibody-treated group (Fig. 3g and h). However, no significant difference in the expression of *jak* (Fig. 3b), *stat* (Fig. 3c), *pgrp* (Fig. 3d), *tak* (Fig. 3e) and toll (Fig. 3f) genes was evident between EL-6 antibody treated and control-antibody-treated *A. phagocytophilum*-infected tick cells. In addition, no significant (*P* > 0.05) difference in the expression of tested immune genes was evident between EL-2 antibody-treated or control-antibody-treated *A. phagocytophilum*-infected tick cells (Supplementary Fig. 2b-h). To further confirm if Toll pathway is critical for EL6 antibody-mediated bacterial clearance, we silenced *myd88* expression in *A. phagocytophilum*-infected tick cells (Fig. 3i). RNAi-mediated knockdown of *myd88* in *A. phagocytophilum*-infected tick cells resulted in significantly (*P* < 0.05) increased bacterial burden (Fig. 3j). However, treatment of *myd88*-dsRNA-containing *A. phagocytophilum*-infected tick cells with EL-6 antibody resulted in significantly (*P* < 0.05) increased *myd88* expression (Fig. 3k) and reduced bacterial burden (Fig. 3l) compared to the levels noted in control-antibody-treated cells. Taken together, these results indicate that EL-6 antibody-treatment efficiently clears *A. phagocytophilum* infection in tick cells through activation of the arthropod innate immune pathway.

### High dose of EL-2 or EL-6 antibodies have cytotoxic effects on tick cells but not on human cells

**Fig. 4:**
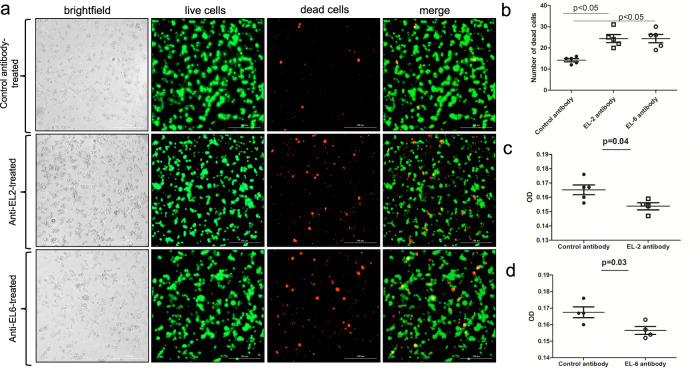
High dose of EL-2 and EL-6 antibody treatment has cytotoxic effects on tick cells. **a** Phase contrast or fluorescent microscopic images showing live (green) or dead (red) uninfected tick cells. Uninfected tick cells were treated with either control or EL-2 or EL-6 antibodies at 10 µg/ml concentration. After 24 h post-treatment, cells were processed for Live/Dead staining assay, followed by imaging using fluorescent microscope. Scale bar indicates 200μm. **b** Quantification of dead cells (red) from 5 images is shown. MTT assay showing viability of tick cells upon treatment with control (**c** and **d**) or EL-2 (**c**) or EL-6 (**d**) antibodies at 10 µg/ml concentration. Y-axis indicates absorbance value obtained by subtracting absorbance measured at 690 nm from absorbance measured at 570 nm. Error bars indicate +/−standard error of the mean. *p* value from student test is shown.

**Fig. 5:**
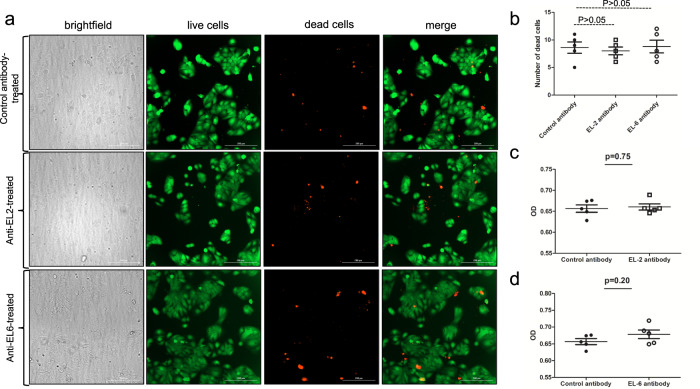
High dose of EL-2 and EL-6 antibody treatment has no cytotoxic effects on human keratinocyte cell line (HaCaT cells). **a** Phase contrast or fluorescent microscopic images showing live (green) or dead (red) uninfected HaCaT cells. Uninfected HaCaT cells were treated with either control or EL-2 or EL-6 antibodies at 10 µg/ml concentration. After 24 h post treatment, cells were processed for Live/Dead staining assay, followed by imaging using fluorescent microscope. Scale bar indicates 200μm. **b** Quantification of dead cells (red) from 5 images is shown. MTT assay showing viability of HaCaT cells upon treatment with control (**c** and **d**) or EL-2 (**c**) or EL-6 (**d**) antibodies at 10 µg/ml concentration. Error bars indicate +/− standard error of the mean. *p* value from student test is shown. Y-axis indicates absorbance value obtained by subtracting absorbance measured at 690 nm from absorbance measured at 570 nm.

To further analyze whether EL-2 or EL-6 antibodies have any cytotoxic effects, we performed Live/Dead cell-based assay as described^38^ with tick cells (Fig. 4) and with human keratinocyte cell line (HaCaT cells) (Fig. 5). Tick (Fig. 4) or HaCaT (Fig. 5) cells were treated with 10 µg/ml of EL-2 or EL-6 antibodies for 24 h and processed for Live/Dead staining and MTT assay. Increased death was observed in tick cells treated with EL-2 or EL-6 antibodies in comparison to the cell death observed upon control antibody treatment (Fig. 4a). Quantification from microscopic images showed significant number of dead cells in EL-6 and EL-2 antibody-treated tick cells compared to the control (Fig. 4b). Furthermore, MTT assay confirmed significantly reduced tick cell viability upon treatment with EL-2 (Fig. 4c) or EL-6 (Fig. 4d) antibodies in comparison to the viability observed in cells treated with control antibody (Fig. 4). However, treatment of HaCaT cells with EL-2 or EL-6 antibodies did not result in increased cell death in comparison to the cell death observed upon control antibody treatment (Fig. 5a). Quantification from microscopic images showed no differences in the number of dead cells between EL-6 and EL-2 antibody-treated tick cells compared to the control (Fig. 5b). MTT assay further confirmed that treatment with EL-2 (Fig. 5c) or EL-6 (Fig. 5d) antibodies did not result in cell death in HaCaT cells. Treatment of tick cells with 5 µg/ml of EL-2 or EL-6 antibodies did not result in considerable cell death (Supplementary Figs. 3 and 4). These results show that EL-2 and EL-6 antibodies are only cytotoxic to tick cells but not to human cells and the effect of these antibodies on cell death was dose dependent.

### Passive immunization of mice with EL-6 antibody affects *A. phagocytophilum* transmission from ticks to the vertebrate host

**Fig. 6:**
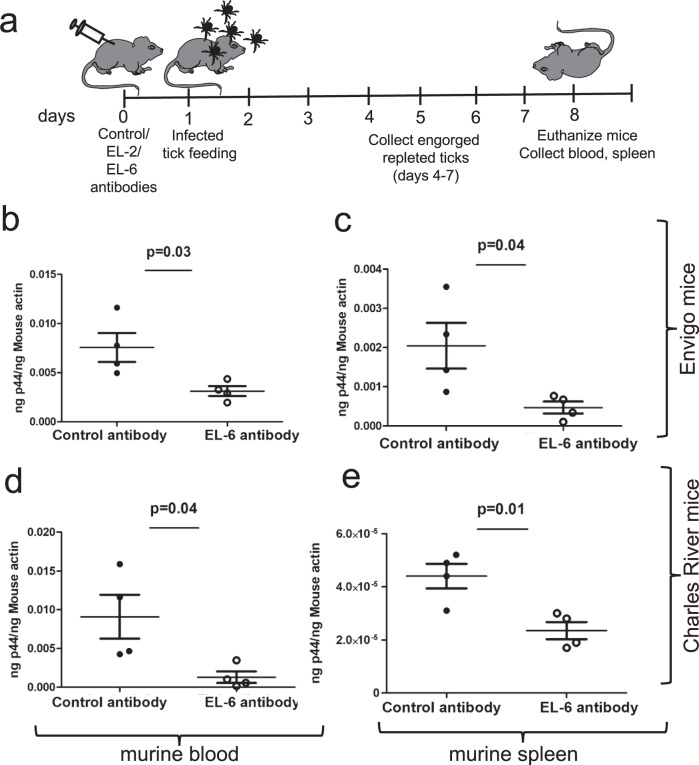
Passive immunization of mice with EL-6 antibody affects tick-mediated transmission of *A. phagocytophilum*. **a** Schematic representation showing plan for the immunization studies. At day 0, mice were passively immunized with control, EL-2 or EL-6 antibodies (15 µg/mouse). At day 1, *A. phagocytophilum*-infected ticks were fed on immunized mice. At days 4-7, repleted and engorged nymphs were collected. At day 8 post-immunization, all mice were euthanized, and blood/spleen tissues were collected. QRT-PCR analysis showing *A. phagocytophilum* burden in control or EL-6 immunized murine blood (**b** and **d**) or spleen tissue (**c** and **e**). Immunizations were performed in mice obtained from Envigo (**b** and **c**) or Charles River Laboratories (**d** and **e**). Bacterial loads were normalized to murine actin levels. Each circle represents data obtained from one mouse sample. Error bars indicate +/− standard error of the mean. *p*-value from student test is shown.

The observation of significantly reduced bacterial burden in tick cells upon treatment with EL-6 antibody (Fig. 3) prompted us to investigate whether passive immunization of a murine host affects *A. phagocytophilum* transmission from ticks to the vertebrate host during blood feeding. Age and background matched C3H mice (C3H/HeNHsd and C3H/HeN) obtained from two different commercial vendors (Envigo and Charles River Laboratories, respectively) were injected with control-antibody or EL-2, or EL-6 antibodies at 15 µg/mouse (Fig. 6 and Supplementary Fig. 5). After 24 h post immunization, *A. phagocytophilum*-infected unfed nymphs (10 ticks/mouse) were fed on these immunized mice (Fig. 6a). Bacterial burden was analyzed in murine blood and spleen tissue on day seven post-tick-placement and in engorged repleted ticks (Fig. 6 and Supplementary Fig. 5). QRT-PCR analysis revealed that *A. phagocytophilum* burden was significantly (*P* < 0.05) reduced in the C3H/HeNHsd (Envigo) mice blood (Fig. 6b) and spleen tissue (Fig. 6c) isolated from EL-6 antibody-immunized mice compared to the bacterial burden noted in the murine blood (Fig. 6b) and spleen tissue (Fig. 6c) isolated from control-IgG-immunized mice. Similar observation was evident with immunization studies performed with C3H/HeN (Charles River Laboratories) mice immunized with EL-6 antibody (Fig. 6d and e). Immunization studies performed with EL-2 antibody did not show any significant differences in the bacterial burden between EL-2 antibody-immunized or control-IgG-antibody-immunized C3H/HeNHsd mice (Supplementary Fig. 5). These results show that immunization with EL-6 antibody effectively reduces transmission of *A. phagocytophilum* from ticks to the murine host.

### Ingestion of EL-6 antibody with the blood meal results in the reduction of *A. phagocytophilum* loads in an engorged tick

**Fig. 7:**
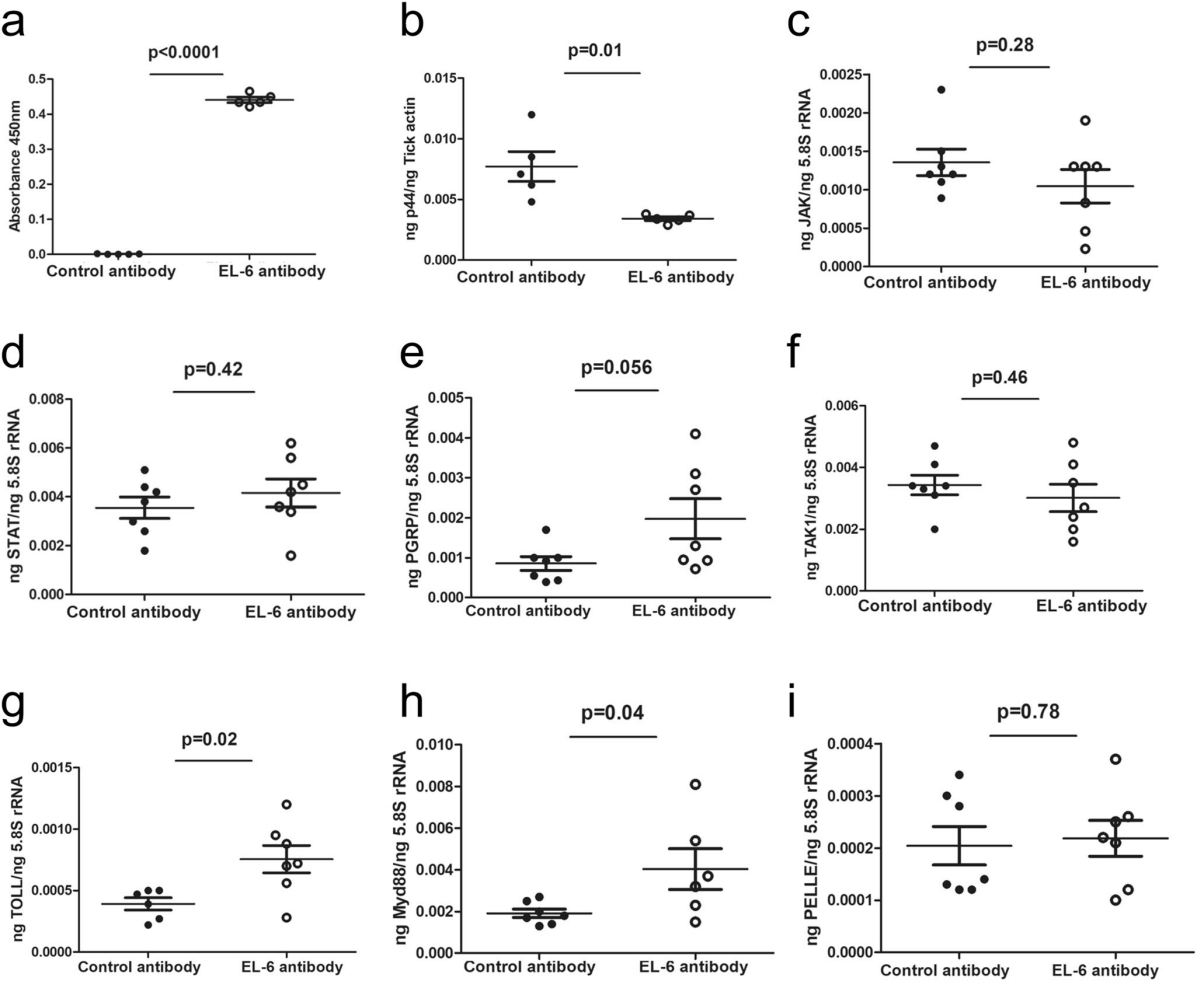
EL-6 antibody affects bacterial loads and induces Toll pathway in engorged ticks. **a** ELISA showing presence of EL-6 antibody in tick body. QRT-PCR analysis showing bacterial burden (**b**) and *jak* (**c**), *stat* (**d**), *pgrp* (**e**), *tak* (**f**), *toll* (**g**), *myd88* (**h**) or *pelle* (**i**) expression in *A. phagocytophilum*-infected ticks fed on control or EL-6 antibody-immunized mice. Mice were immunized with control or EL-6 antibodies at a concentration of 15 µg/mouse. *A. phagocytophilum* p44 levels were normalized to tick actin levels and mRNA levels of tick innate immune genes were normalized to tick 5.8 S rRNA levels. *p*-value from student test is shown. Error bars indicate +/− standard error of the mean. Each circle represents data obtained from the sample generated from one tick.

To determine the effect of passive immunization of the murine host on the *A. phagocytophilum* burden within tick itself, samples from engorged repleted ticks were processed for PCRs and ELISA (Fig. 7 and Supplementary Fig. 6). ELISA showed that *A. phagocytophilum*-infected nymphs ingested EL-6 (Fig. 7a) and EL-2 (Supplementary Fig. 6a) antibodies into its body with the blood meal. QRT-PCR analysis showed that *A. phagocytophilum* burden was significantly (*P* < 0.05) reduced in ticks fed on EL-6 antibody-immunized mice compared to the burden noted in ticks fed on control antibody-immunized mice (Fig. 7b). However, no significant difference in the bacterial burden was evident between ticks fed on EL-2 antibody or control-antibody-immunized mice (Supplementary Fig. 6b). No significant differences in the expression of *jak, stat, pgrp, tak1 and pelle* were evident in ticks fed on EL-6 antibody or control-antibody-immunized mice (Fig. 7). However, arthropod *toll* transcripts were significantly upregulated in ticks fed on EL-6 antibody immunized mice compared to the levels noted in ticks fed on control antibody-immunized animals (Fig. 7g). In addition, like the observation noted with tick cells, arthropod *myd88* transcripts were significantly upregulated in ticks fed on EL-6 antibody immunized mice compared to the levels noted in ticks fed on control antibody-immunized animals (Fig. 7h). In addition, we did not observe any significant differences in the expression of any of the analyzed arthropod immune genes in ticks fed on EL-2 antibody immunized mice compared to the levels noted in ticks fed on control antibody-immunized mice (Supplementary Fig. 6). Collectively, these results show that EL-6 antibody results in the reduction in the bacterial loads in an engorged tick due to activation of arthropod immune pathways mediated by Myd88.

### Ingestion of EL-6 antibody with the blood meal adversely affects tick molting

**Fig. 8:**
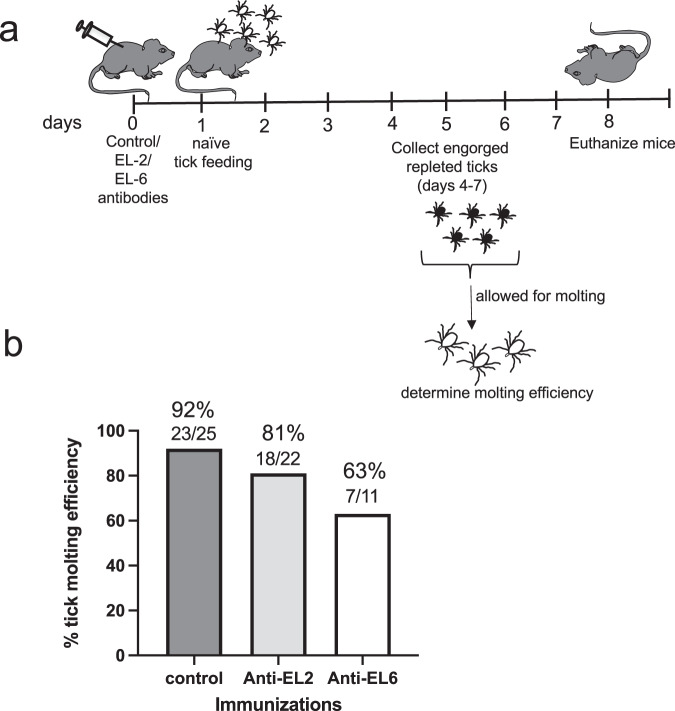
EL-6 antibody affects molting in ticks. **a** Schematic representation showing plan for the immunization studies. On day 0, mice were passively immunized with control, EL-2 or EL-6 antibodies (30 µg/mouse). On day 1, uninfected ticks were fed on immunized mice. On days 4-7, repleted and engorged nymphs were collected. On day 8 post-immunization, all mice were euthanized. Engorged nymphs were incubated in an environmental chamber to allow ticks to molt. **b** Histogram showing percentage of molting efficiency of ticks fed on control or EL-2 or EL-6 antibody immunized mice. The number of ticks molted to total number analyzed in this study are indicated on the top of each bar.

The observation of tick cell death upon treatment with EL-2 or EL-6 antibodies prompted us to investigate whether these antibodies affect tick molting. Age and background matched C3H/HeNHsd (Envigo) were injected with control-antibody or EL-2, or EL-6 antibodies at 30 µg/mouse (Fig. 8a). After 24 h postimmunization, naïve unfed nymphs (15 ticks/mouse) were fed on these immunized mice (Fig. 8a). Repleted engorged ticks were collected from days 3–5 post tick placement. A total of 25, 22 and 11 fed nymphs were collected from control-antibody, EL-2 or EL-6 antibody-immunized mice, respectively. These fed nymphal ticks were housed in an environmental chamber for molting (Fig. 8a). Fed nymphal ticks started molting to adult stage after 30 days of incubation. We noted that 92% of nymphal ticks that were fed on control-antibody-immunized mice molted to adults (Fig. 8b). Whereas 81% or 63% of nymphal ticks fed on EL-2 or EL-6 antibody-immunized mice, respectively, molted to adults (Fig. 8b). Collectively, these results show that treatment with EL-6 antibody not only causes tick cell death in vitro but also affects tick molting.

## Discussion

Development of an anti-tick vaccine targeting arthropod protective antigens is an innovative approach in the field of vaccinology^6,18,22,23,45^. It offers to target more than one pathogen transmitted by the same tick^6,18,22,23,45^. Anti-vector vaccine strategies are designed to block acquisition of pathogen into ticks or colonization in ticks, or transmission of pathogens from the ticks^6,18,22,23,45^. Several studies have tested or proposed to generate vaccines targeting tick secreted, cytosolic or membrane proteins^6,18,21–23,45^. In this study, we provide evidence that targeting tick IsOATP4056, a membrane transporter protein, affects the transmission of *A. phagocytophilum* from the arthropod vector to the vertebrate host.

The results from ELISA, immunoblotting and immunofluorescence analyses that show increased levels of IsOATP4056 in *A. phagocytophilum*-infected ticks and tick cells compared to the levels noted in uninfected controls are in line with the gene expression data previously published from our laboratory^35,37,38,46^. Based on the primary amino acid sequence, the expected molecular weight of the protein is approximately 100 kDa. IsOATP4056 has several posttranslational modification residues including glycosylation sites^36^. Immunoblotting with EL-2 and EL-6 antibodies in the absence of deglycosylation mix showed an intense band at >250 kDa in all *A. phaghocytophilum*-infected tick and tick cell samples. The presence of increased intense band (detected with EL2 antibody) in infected tick cell sample > 250 kDa even after deglycosylation indicates that IsOATP4056 could be present in an oligomerized form or in other posttranslational-modified form. The detection of band ~ 250 kDa indicates dimerization of IsOATp4056 and the band at ~ 100 kDa indicates monomeric form. EL-2 and EL-6 are different extracellular loops of IsOATP4056 protein. Deglycosylation did not impact binding of EL-6 antibody to the oligomerized form or other posttranslational-modified form of IsOATP4056 (band >250 kDa). We believe that the binding affinity for EL-2 and EL-6 antibodies on IsOATP4056 could be different. Where, EL-6 antibody could readily recognize the epitope in the oligomeric form of IsOATP4056 and EL-2 antibody could recognize oligomeric/other post-translational-modified form more but also could recognize deglycosylated/dimeric/monomeric form of IsOATP4056. The observation of multiple forms of tick IsOATP4056 is consistent with the observation of mammalian OATPs that forms dimers/homo or hetero-oligomers^47,48^. In addition, observation of multiple bands in immunoblotting analyses with EL-2 antibody suggests that IsOATP4056 might also be proteolytically cleaved.

In a recent study, we reported a circadian control of Toll and JAK/STAT pathway immune genes such as *pelle* and *jak*, respectively, are critical for *A. phagocytophilum* transmission from ticks to the vertebrate host^44^. In this study, we noted that activation of Toll pathway, in particular Myd88, is critical for bacterial reduction in ticks and tick cells in the presence of EL-6 antibody. When either EL-2 or EL-6 antibody was used for the treatment of ticks or tick cells, only EL-6 antibody-treatment resulted in the activation of Toll/Myd88 pathway that perhaps led to the reduction in the bacterial loads. The observation of significantly increased bacterial burden in *myd88*-dsRNA-treated tick cells followed by the reduction in the bacteria in these cells upon EL-6 antibody treatment further confirmed EL-6-mediated activation of Toll pathway in bacterial clearance. IsOATP4056 has six loops, where EL-2 antibody binds on the epitope on the second loop and EL-6 antibody binds epitope on the sixth loop. We believe that binding of EL-6 antibody on the sixth loop might have resulted in the perturbation of signaling that eventually resulted in the activation of Toll pathway in ticks. However, the detailed mechanism of Toll pathway activation following the EL-6 antibody binding on IsOATP4056 needs to be elucidated. In this context, it is interesting to note that a previous study reported downregulation of mouse OATP-4 in a TLR-4-dependent manner upon treatment with LPS^49^.

The observation of cell death in tick cells and not in HaCaT cells upon treatment with 10 µg/ml of EL-2 or EL-6 antibodies suggests that these antibodies are specific to tick IsOATP4056. However, this observation was dependent on the dose of the antibody as no cell death was noted in tick cells upon treatment with 5 µg/ml. OATPs participate in the transport of several molecules^39–43^. Therefore, it is not surprising to hypothesize that blocking extracellular loops of IsOATP4056 could affect the transport of various molecules, including nutrients that eventually could lead to cell death. In addition, the observation of reduced molting in ticks fed on EL-6 antibody-immunized mice further supports that IsOATP4056 is critical for tick cell survival.

Ticks ingest host proteins in a blood meal upon feeding on a vertebrate host^50^. Therefore, it is reasonable to hypothesize that ticks could also ingest antibodies during feeding on EL-6 or EL-2 antibody-immunized mice. Our ELISA data clearly shows that ticks do ingest both EL-6 and EL-2 antibodies in a blood meal. The reduction in the bacteria loads in ticks fed on EL-6 immunized mice indicates that targeting IsOATP4056 could also be envisioned as an ideal therapeutic approach to impair transstadial transmission of *A. phagocytophilum* from one tick developmental stage to other.

The observation of reduced bacterial loads in blood and spleen tissue in mice (obtained from two different commercial vendors) immunized with EL-6 antibody but not with EL-2 antibody clearly shows that blocking arthropod IsOATP4056 at EL-6 region impairs *A. phagocytophilum* transmission from ticks to the vertebrate murine host. Several scenarios can be envisioned where EL-6 region could play an important role in IsOATP4056 function in ticks. First, EL-6 could be facilitating bacterial exit from the cells and blocking this region could affect *A. phagocytophilum* transmission. Second, EL-6 region could repress Toll pathway signaling and blocking this region on IsOATP4056 could activate TOLL pathway and lead to reduction in the bacterial loads. Third, EL-6 region could be involved in XA transport. XA is critical for *A. phagocytophilum* colonization in ticks and tick cells^35,37^. Therefore, blocking EL-6 region could affect *A. phagocytophilum* survival and exit from tick cells. With any or all these hypotheses, blocking IsOATP4056 at EL-6 region seems to be an effective approach to impair the transmission of *A. phagocytophilum* from ticks to the vertebrae host.

In summary, this study provides evidence that targeting arthropod membrane transporter IsOATP4056 affects *A. phagocytophilum* transmission from ticks to the vertebrate host. Studies such as these are not only important to understand the role of tick molecules in vector-pathogen interactions but also lead us in the development of anti-vector vaccine to target transmission of *A. phagocytophilum* and perhaps other pathogens from medically important tick vector to the vertebrate host.

## Methods

### Bacterial isolates, ticks, and tick cell line

*Anaplasma phagocytophilum* strain NCH-1 was used throughout this study. This strain was obtained from BEI Resources, NIAID, NIH. *Anaplasma phagocytophilum* was maintained in the human promyelocytic cell line (HL-60 cells) as described in our previous studies^35,38,44^. The HL-60 cell line was obtained from ATCC, USA. *Ixodes scapularis* larvae were obtained from BEI Resources, NIAID, NIH. Transmission studies were performed with molted unfed *A. phagocytophilum*-infected nymphs. The tick cell line, ISE6, obtained from ATCC, USA, was used throughout this study. Tick cell line was maintained as described in our previous studies^35,38,44^. Tick rearing was conducted in an Environmental Chamber from Parameter Generation and Control, USA. The incubator was set at 23 ± 2 °C with 94% relative humidity and 14:10 light:dark conditions.

### Antibody generation

IsOATP4056 polyclonal antibodies used in this study were generated at a commercial facility (GenScript, USA). The peptides corresponding to IsOATP4056 regions of extracellular loops 2 (NVTRIEDENTCQMP) and 6 with 3’ cysteine (TWRHKRELKEAPPLC) were used to generate EL-2 and EL-6 antibodies, respectively. Antibodies were sterile-filtered and had no preservatives. Upon receipt of the antibodies, control, EL-2 or EL-6 antibodies were solubilized in water and used for animal experiments. The volume for 15 or 30 ug/mouse of EL-2 or EL-6 or control-IgG antibody solutions were adjusted with 0.9% sodium chloride to 100 µl injection volume.

### Mice, tick feeding and immunizations

C3H/HeN (female mice, 4–6 weeks old, CharlesRiver Laboratories, USA) and C3H/HeNHsd (female mice, 4–6 weeks old, Envigo) was used in this study. B6.129S7-Rag1tm1Mom/J mice (Jackson Laboratories, USA) was used to maintain *A. phagocytophilum* infection. Larval ticks were fed on uninfected or *A. phagocytophilum*-infected mice and molted to nymphs. Unfed *A. phagocytophilum*-infected nymphs were fed on control, EL2 or EL6-antibody-immunized mice. Briefly, groups of four C3H/HeN or C3H/HeNHsd mice were injected (intraperitoneal, i.p.) with 15 µg/mouse of control, EL-2 or EL-6 antibodies one day prior to the placement of ticks. The schematic description of immunization experiment is provided as a panel in main figures. After 1 day postimmunization, 10 *A. phagocytophilum*-infected ticks were fed on each immunized mouse. Engorged ticks were collected after repletion and processed for RNA, DNA, or protein extractions to analyze tick innate immune gene expression, bacterial burden, and ELISA to determine the intake of antibodies, respectively. Mice were euthanized on day 7 post tick placement (day 8 postimmunization) and blood and spleen tissues were collected and processed for DNA extractions to evaluate bacterial burden. In the second set of experiments, groups of four C3H/HeNHsd mice/group were immunized with control, EL-2 or EL-6 antibodies (30 µg/mouse) and after day 1 postimmunization, 15 uninfected nymphs were fed on each mouse. Engorged ticks were collected after repletion and incubated in an environmental chamber for evaluating molting efficiency. The percentage of molting was determined based on the number of molted adults to the number of engorged nymphs that were incubated for molting.

### Ethics statement

All animal work in this study was performed based on the approved animal protocol from the University of Tennessee Institutional Animal Care and Use Committee (IACUC) with permit number 2801-0221. Animal experiments were performed in accordance with the Guide for the Care and Use of Laboratory Animals of the National Institute of Health. To minimize distress in animals during tick feeding, acepromazine was used as a tranquilizer.

### Cell culture, bacteria and in vitro infection

Tick cell line ISE 6 was maintained in LI5B300 medium as described in our previous studies^35,37^. Briefly, 2 × 10^5^ cells/well were seeded in cell culture plates one day prior to infection. For in vitro tick cell infections, *A. phagocytophilum* was isolated from HL-60 cultures maintained in IMDM medium. Host cell free *A. phagocytophilum* was isolated as described^30,35,51^. Briefly, *A. phagocytophilum*-infected HL-60 cells were centrifuged at 3005 g for 10 min. Cell pellets were resuspended in IMDM medium and incubated in −80 °C freezer for 10 min to allow increased lysis of cells. Cells were passed through a 27-gauge syringe 6-8 times to release the bacteria. The cell debris was pelleted by centrifugation at 270 g for 3 min and the supernatant was collected. For in vitro cell line infections, 2 × 10^5^ /tick cells/well were infected with *A. phagocytophilum* isolated from 4 ×10^5^ infected HL-60 cells in a 12 well cell culture plate. For antibody-blocking assays, 2 × 10^5^ /tick cells/well were plated one day prior to treatment with antibodies. Antibodies (either 5 or 10 µg/ml) were resuspended in 50 µl sterile 1x PBS before adding into the culture wells. After 24 h post antibody treatment, cells were infected with *A. phagocytophilum*. Tick cells were collected after 24 h post infection and processed for RNA and DNA extractions to evaluate gene expression and bacterial burden, respectively.

### Immunoblotting

Immunoblotting was performed as described^30^. Briefly, IsOATP4056 levels were analyzed in uninfected or *A. phagocytophilum*-infected whole tick or tick cell lysates with rabbit polyclonal EL-2 and EL-6 antibodies. Protein lysates generated from uninfected ticks or tick cells and *A. phagocytophilum*-infected ticks or tick cells were derived from same experiment and processed in parallel. SDS PAGE with 8% resolving gel was used and blotting was performed using Trans-Blot Turbo^TM^ (BioRad, USA) by setting the parameters as Voltage-25V, Current-2.5 A and time-7 min Blotted nitrocellulose membranes were blocked with 5% BSA in TBST (0.05% Tween 20) for 1 hr at room temperature and incubated overnight in primary antibody solution at a dilution of 1: 1000 which was prepared in 5% BSA, dissolved in TBST. Full images of blots/Coomassie staining/Ponceau staining are shown in the Supplementary Fig. 7. Anti-rabbit secondary antibody conjugated to horse radish peroxidase (Cell Signaling Technologies, USA) was used at a dilution of 1: 5000 and with incubation for 1 hr at room temperature. Chemiluminescence was measured using Clarity^TM^ Western ECL substrate, BioRad in Chemidoc^TM^ MP imaging system (BioRad, USA). Deglycosylation of tick cell samples was done with Protein Deglycosylation Mix II (NEB- P6044S) following manufacturer’s instructions. Briefly, 10 µg of tick cell lysate was mixed with 0.5 µl of deglycosylation buffer 2 and incubated at 75 °C for 10 minutes. After cooling, the samples were mixed with 0.5 µl of Protein Deglycosylation Mix II and incubated at room temperature for 30 min followed by incubation at 37 °C overnight and processed for SDS PAGE and immunoblotting.

### Enzyme-Linked Immunosorbent Assay (ELISA)

To determine the specificity of antibodies binding to respective peptides, 100 ng of EL-2 or EL-6 peptides (GenScript, USA) were dissolved in 1x PBS, and coated to wells in a 96-well plate (Nunc, ThermoFisherScientific, USA) and incubated overnight at 4 °C. Scrambled peptide (GenScript, USA) was also coated in a similar way. Empty wells were used for reference. Similarly, 250 ng of uninfected or *A. phagocytophilum*-infected tick or tick cell lysates were coated on to plates and incubated for overnight at 4 °C. All wells were blocked with blocking buffer (0.1% Tween 20 and 1% BSA in 1x PBS) for 1 hr at 37 °C. 500 ng of primary antibodies were added on to each well after making up with 1x PBS containing 0.05% Tween 20 and 0.5% BSA and incubated for 1 hr at 37 °C. After washing 3 times with wash buffer (1x PBS containing 0.05% Tween 20), antirabbit secondary antibody conjugated with horse radish peroxidase was added at a dilution of 1: 2000 and incubated for 1 hr at 37 °C. After washing 3 times, 100 µl of peroxidase substrate (Sure Blue^TM^, KPL, USA) was added and incubated for 15 min at 37 °C. Subsequently, 50 µl of stop solution was added and absorbance was measured at 450 nm in CYTATION7 (BioTek, USA). For detection of antibodies in fed tick nymphs, total protein lysates from ticks fed on EL-2 or EL-6 or control antibody-immunized mice were generated in 1x PBS buffer containing 1 mM PMSF. Five micrograms of each of these lysates were added to EL-2 or EL-6 peptide-coated 96 well plates. After 24 h incubation at 4 °C, plates were processed for secondary antibody treatment followed by substrate addition and absorbance reading at 450 nm.

### DNA, RNA extractions and quantitative real-time PCR (QRT-PCR)

QRT-PCR was performed as described in our previous publications^35,44^. *Anaplasma phagocytophilum* loads in tick cells, fed nymphal ticks or mouse samples were measured by taking the ratios of absolute amount of bacterial *p44* gene copies and that of respective actin gene copies in host cells. Standard curves for QRT-PCR for each gene fragment were prepared starting from 1 ng to 0.00001 ng/ul. DNA was extracted using DNeasy blood and tissue extraction kit (QIAGEN, USA). Transcript loads of selected innate immune genes of ticks were expressed as the ratios of absolute amounts of individual transcripts to that of tick 5.8 S rRNA. Total RNA was extracted using Aurum Total RNA Mini kit (BioRad, USA) and cDNA was prepared using iSCRIPT cDNA synthesis kit (BioRad, USA). QRT-PCR was performed in CFX Opus 96 (BioRad, USA) using iQ-SYBR Green Supermix (BioRad, USA) or 2X Universal SYBR Green fast qPCR Mix (ABclonal, USA). Following are the oligonucleotides used to measure transcript of tick *pgrp*: 5’ CGGCTACACGAGACCTTGCT 3’ and 5’ CGCGACGTGACTGGGGT 3’. For other genes, previously published oligonucleotides were used^35,38,44^.

### Immunofluorescence

Tick cells (2 × 10^4^) were plated in each well of a 96 black transparent-bottom plate (Griener Bio One, ThermoFisherScientific, USA). After 24 h postplating, *A. phagocytophilum* was infected in one group of tick cells. After 24 h post-infection, uninfected and *A. phagocytophilum*-infected tick cells were processed for immunofluorescence. To localize IsOATP4056, uninfected or *A. phagocytophilum*-infected tick cells were fixed in 4% paraformaldehyde, permeabilized in 0.2% Triton X-100 and blocked with 3% BSA. EL-6 antibody was used at 1:250 dilution followed by treatment with anti-rabbit-alexa594-conjugated secondary antibody (Molecular Probes, ThermoFisher Scientific, USA) at a dilution of 1: 2500. Cells were further stained with DAPI (Invitrogen, ThermoFisher Scientific, USA) and images were captured with 40X objective in CYTATION7 imager (BioTek, USA).

### Live/Dead and MTT assay

Live/Dead and MTT assays were performed as described in our previous publications^46,52^. Briefly, 3 × 10^4^ tick cells were plated in each well of a 96 well plate (Griener Bio One, ThermoFisherScientific, USA). After one day of plating, cells were treated with 5 µg/ml or 10 µg/ml of EL-2 or EL-6 antibodies. After one day of antibody treatment, cell death assays were performed. In the case of Live/Dead assay (LIVE/DEAD^TM^ Cell Imaging kit, Invitrogen), the contents of green and red vials were mixed as per the instructions and added in the culture medium at ¼ dilution. After 15 min of incubation, cells were imaged for fluorescence using 20X objective in CYTATION7 imager (BioTek, USA). In the case of MTT assay, 5 mg/ml of MTT reagent (Sigma, USA) in 1x PBS was added to cells at 10% of the total volume. Plates were incubated at 37 °C for 4 h and DMSO was added at half the volume of culture medium. After mixing for 5 min, absorbance at 570 nm and 690 nm were measured. Values at 690 nm were subtracted from the respective values at 570 nm and graphs were plotted.

### dsRNA synthesis and tick cell line experiment

The mock- or *myd88*-dsRNA synthesis was performed as described^35,38^. Briefly, *myd88*-dsRNA fragment was generated by PCR using 5’ CCAGATCTGTCCATCAAGAGCAGTGGCA 3’ and 5’ CGGGTACCACCATTGTGAAGGAGCACCA 3’ containing BglII and KpnI sites, respectively. The obtained PCR product was cloned in to pL4440 double T7 Script II vector as described^29,30^. The obtained clone was later processed for dsRNA synthesis using MEGAscript RNAi Kit (Ambion Inc.) following manufacturer’s instructions. Transfection of *myd88*-dsRNA into ISE6 tick cells was performed with 2 microliters of FuGENE 6 transfection reagent (Promega, USA) as described^35,38^. Briefly, 2 ×10^5^ cells were plated in a 12-well plate in L15B300 medium. After overnight incubation, 750 ng of *myd88* dsRNA was mixed with FuGENE 6 transfection reagent and was added to each well. After 4 hr, 2x L15B300 medium was added with or without EL-6 antibody and incubated overnight. Following incubation, cell free *A. phagocytophilum* was added and samples were collected after 24 h post infection. Samples were processed for RNA and DNA extraction to quantify *myd88* expression and bacterial burden, respectively.

### Statistics

All the data sets were statistically analyzed using GraphPad Prism 5 software (www.graphpad.com). The non-paired Student *t*-test was considered to compare the experimental data sets with two variables. *p* values of <0.05 are considered as significant.

### Reporting summary

Further information on research design is available in the Nature Research Reporting Summary linked to this article.

## Supplementary information

Supplementary information

REPORTING SUMMARY

## Data Availability

All data generated or analyzed during this study are included in this published article (and its supplementary information files).

## References

[1] Rochlin I, Toledo A (2020). Emerging tick-borne pathogens of public health importance: a mini-review. J. Med. Microbiol..

[2] Steere AC (2016). Lyme borreliosis. Nat. Rev. Dis. Prim..

[3] Patel M (2021). Emerging and re-emerging viral infections in India. J. Prev. Med. Hyg..

[4] Rodino KG, Theel ES, Pritt BS (2020). Tick-Borne Diseases in the United States. Clin. Chem..

[5] Zhao GP (2021). Mapping ticks and tick-borne pathogens in China. Nat. Commun..

[6] Neelakanta G, Sultana H (2015). Transmission-Blocking Vaccines: Focus on Anti-Vector Vaccines against Tick-Borne Diseases. Arch. Immunol. Ther. Exp. (Warsz.).

[7] Ribeiro CM (2021). Prevalence of Rickettsia rickettsii in Ticks: Systematic Review and Meta-Analysis. Vector Borne Zoonotic Dis..

[8] Gilbert L (2021). The Impacts of Climate Change on Ticks and Tick-Borne Disease Risk. Annu Rev. Entomol..

[9] Mlera L, Bloom ME (2018). The Role of Mammalian Reservoir Hosts in Tick-Borne Flavivirus Biology. Front Cell Infect. Microbiol.

[10] Anderson JF, Magnarelli LA (2008). Biology of ticks. Infect. Dis. Clin. North Am..

[11] Kunz SE, Kemp DH (1994). Insecticides and acaricides: resistance and environmental impact. Rev. Sci. Tech..

[12] Pugh, S. J. et al. Effectiveness of two doses of tick-borne encephalitis (TBE) vaccine. *J. Travel Med*. **29**, 10.1093/jtm/taab193 (2022). 10.1093/jtm/taab193 34999897

[13] de la Fuente J, Contreras M (2015). Tick vaccines: current status and future directions. Expert Rev. Vacc.

[14] Smit R, Postma MJ (2016). Vaccines for tick-borne diseases and cost-effectiveness of vaccination: a public health challenge to reduce the diseases’ burden. Expert Rev. Vacc..

[15] Moutailler S (2016). Co-infection of Ticks: The Rule Rather Than the Exception. PLoS Negl. Trop. Dis..

[16] Civitello DJ, Rynkiewicz E, Clay K (2010). Meta-Analysis of Co-Infections in Ticks. Isr. J. Ecol. Evol..

[17] Nieto NC (2018). Using citizen science to describe the prevalence and distribution of tick bite and exposure to tick-borne diseases in the United States. PLoS One.

[18] de la Fuente J, Kopacek P, Lew-Tabor A, Maritz-Olivier C (2016). Strategies for new and improved vaccines against ticks and tick-borne diseases. Parasite Immunol..

[19] Merino O, Alberdi P, Perez de la Lastra JM, de la Fuente J (2013). Tick vaccines and the control of tick-borne pathogens. Front Cell Infect. Microbiol.

[20] de la Fuente J (2011). Targeting arthropod subolesin/akirin for the development of a universal vaccine for control of vector infestations and pathogen transmission. Vet. Parasitol..

[21] Neelakanta G, Sultana H (2021). Tick Saliva and Salivary Glands: What Do We Know So Far on Their Role in Arthropod Blood Feeding and Pathogen Transmission. Front Cell Infect. Microbiol.

[22] van Oosterwijk JG (2021). Anti-tick and pathogen transmission blocking vaccines. Parasite Immunol..

[23] Ndawula, C. Jr. & Tabor, A. E. Cocktail Anti-Tick Vaccines: The Unforeseen Constraints and Approaches toward Enhanced Efficacies. *Vaccines (Basel)* **8**, 10.3390/vaccines8030457 (2020). 10.3390/vaccines8030457 PMC7564958 32824962

[24] Narasimhan S (2021). Acquired tick resistance: The trail is hot. Parasite Immunol..

[25] Bakken JS, Dumler JS (2015). Human granulocytic anaplasmosis. Infect. Dis. Clin. North Am..

[26] Dumler JS (2005). Human granulocytic anaplasmosis and Anaplasma phagocytophilum. Emerg. Infect. Dis..

[27] Carlyon JA, Fikrig E (2003). Invasion and survival strategies of Anaplasma phagocytophilum. Cell Microbiol.

[28] Rikihisa Y (2011). Mechanisms of obligatory intracellular infection with Anaplasma phagocytophilum. Clin. Microbiol. Rev..

[29] Neelakanta G, Sultana H, Fish D, Anderson JF, Fikrig E (2010). Anaplasma phagocytophilum induces Ixodes scapularis ticks to express an antifreeze glycoprotein gene that enhances their survival in the cold. J. Clin. Invest.

[30] Sultana H (2010). Anaplasma phagocytophilum induces actin phosphorylation to selectively regulate gene transcription in Ixodes scapularis ticks. J. Exp. Med.

[31] de la Fuente J (2017). Tick-Pathogen Interactions and Vector Competence: Identification of Molecular Drivers for Tick-Borne Diseases. Front Cell Infect. Microbiol.

[32] Oliva Chavez AS (2015). An O-Methyltransferase Is Required for Infection of Tick Cells by Anaplasma phagocytophilum. PLoS Pathog..

[33] O’Neal, A. J., Singh, N., Mendes, M. T. & Pedra, J. H. F. The genus Anaplasma: drawing back the curtain on tick-pathogen interactions. *Pathog. Dis.* **79**, 10.1093/femspd/ftab022 (2021). 10.1093/femspd/ftab022 PMC8062235 33792663

[34] Rosche KL, Sidak-Loftis LC, Hurtado J, Fisk EA, Shaw DK (2020). Arthropods Under Pressure: Stress Responses and Immunity at the Pathogen-Vector Interface. Front Immunol..

[35] Taank V (2017). Human rickettsial pathogen modulates arthropod organic anion transporting polypeptide and tryptophan pathway for its survival in ticks. Sci. Rep..

[36] Taank V (2018). Characterization of tick organic anion transporting polypeptides (OATPs) upon bacterial and viral infections. Parasit. Vectors.

[37] Dahmani M, Anderson JF, Sultana H, Neelakanta G (2020). Rickettsial pathogen uses arthropod tryptophan pathway metabolites to evade reactive oxygen species in tick cells. Cell Microbiol..

[38] Ramasamy E, Taank V, Anderson JF, Sultana H, Neelakanta G (2020). Repression of tick microRNA-133 induces organic anion transporting polypeptide expression critical for Anaplasma phagocytophilum survival in the vector and transmission to the vertebrate host. PLoS Genet.

[39] Hagenbuch B, Meier PJ (2004). Organic anion transporting polypeptides of the OATP/ SLC21 family: phylogenetic classification as OATP/ SLCO superfamily, new nomenclature and molecular/functional properties. Pflug. Arch..

[40] Kalliokoski A, Niemi M (2009). Impact of OATP transporters on pharmacokinetics. Br. J. Pharm..

[41] Nigam SK (2015). The organic anion transporter (OAT) family: a systems biology perspective. Physiol. Rev..

[42] Stieger B, Hagenbuch B (2014). Organic anion-transporting polypeptides. Curr. Top. Membr..

[43] Roth M, Obaidat A, Hagenbuch B (2012). OATPs, OATs and OCTs: the organic anion and cation transporters of the SLCO and SLC22A gene superfamilies. Br. J. Pharm..

[44] Khanal, S., Taank, V., Anderson, J. F., Sultana, H. & Neelakanta, G. Rickettsial Pathogen Perturbs Tick Circadian Gene to Infect the Vertebrate Host. *Int. J. Mol. Sci.* **23**, 10.3390/ijms23073545 (2022). 10.3390/ijms23073545 PMC8998576 35408905

[45] de la Fuente J (2021). Translational biotechnology for the control of ticks and tick-borne diseases. Ticks Tick. Borne Dis..

[46] Namjoshi P, Dahmani M, Sultana H, Neelakanta G (2023). Rickettsial pathogen inhibits tick cell death through tryptophan metabolite mediated activation of p38 MAP kinase. iScience.

[47] Lee W (2005). Polymorphisms in human organic anion-transporting polypeptide 1A2 (OATP1A2): implications for altered drug disposition and central nervous system drug entry. J. Biol. Chem..

[48] Zhang Y, Boxberger KH, Hagenbuch B (2017). Organic anion transporting polypeptide 1B3 can form homo- and hetero-oligomers. PLoS One.

[49] Li N, Choudhuri S, Cherrington NJ, Klaassen CD (2004). Down-regulation of mouse organic anion-transporting polypeptide 4 (Oatp4; Oatp1b2; Slc21a10) mRNA by lipopolysaccharide through the toll-like receptor 4 (TLR4). Drug Metab. Dispos..

[50] Vora A (2017). Ticks elicit variable fibrinogenolytic activities upon feeding on hosts with different immune backgrounds. Sci. Rep..

[51] Thomas V, Fikrig E (2007). Anaplasma phagocytophilum specifically induces tyrosine phosphorylation of ROCK1 during infection. Cell Microbiol.

[52] Dutta S (2018). Coordination of different ligands to copper(II) and cobalt(III) metal centers enhances Zika virus and dengue virus loads in both arthropod cells and human keratinocytes. Biochim Biophys. Acta Gen. Subj..

