# Supplementary material for: Immunization against arthropod protein impairs transmission of rickettsial pathogen from ticks to the vertebrate host
Source: NPJ Vaccines. 2023 May 30;8:79. doi: 10.1038/s41541-023-00678-y (PMC10229574; doi:10.1038/s41541-023-00678-y)
Supplement: Supplementary file 1 — Supplementary information [file 41541_2023_678_MOESM1_ESM.pdf]

Immunization against arthropod protein impairs transmission of rickettsial pathogen from ticks to the vertebrate host

P P Mahesh [1], Prachi Namjoshi [1], Hameeda Sultana [1], and Girish Neelakanta [1,*]

Supplementary Information

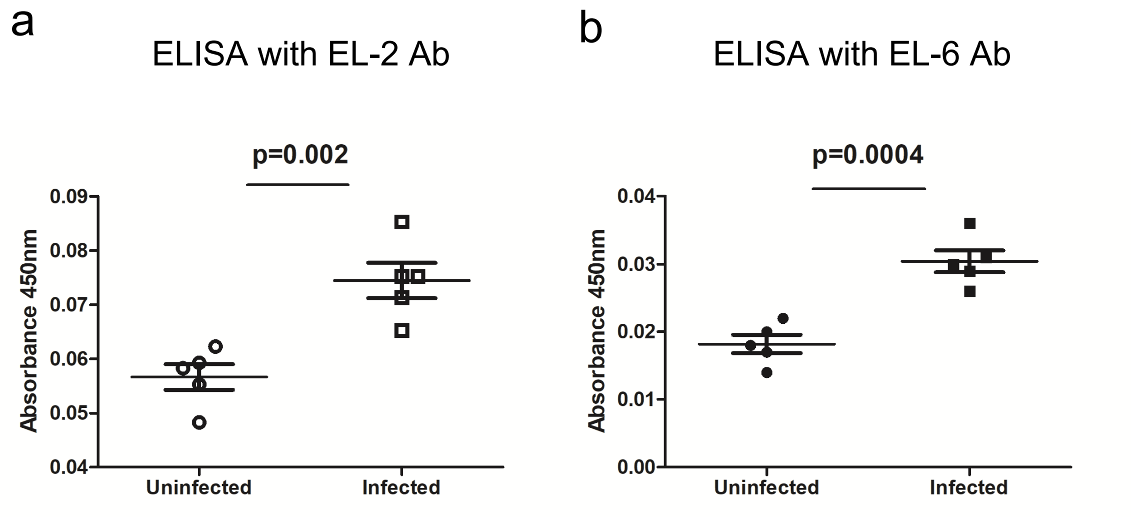

Supplementary Figure 1

Supplementary Figure 1. ***Anaplasma phagocytophilum*** **upregulates IsOATP4056 in tick cells.** ELISA performed with total lysates prepared from uninfected or *A. phagocytophilum*-infected tick cells probed with EL-2 (a) or EL-6 (b) antibody is shown. Each dot represents sample generated from one culture well. Error bars indicate +/- standard error of the mean. P-value from student test is shown.

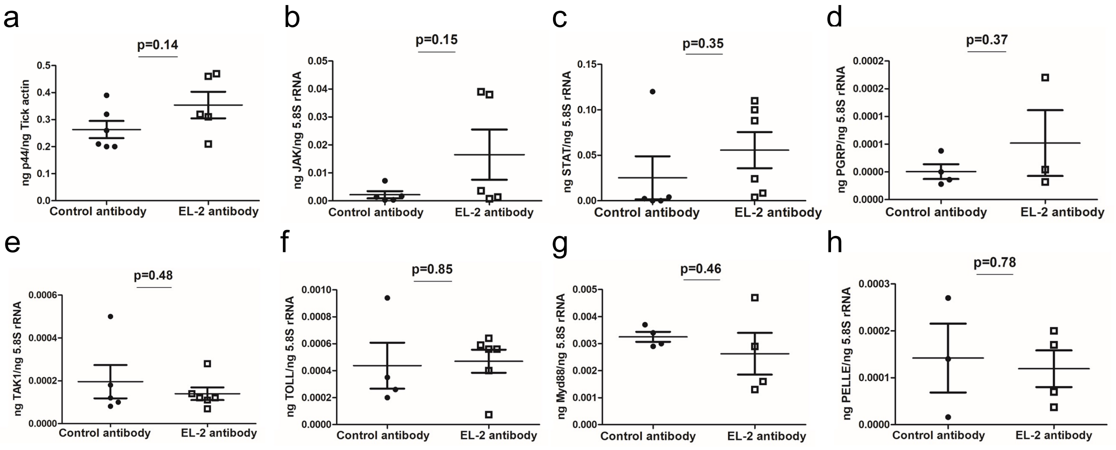

Supplementary Figure 2

Supplementary Figure 2. EL-2 antibody treatment has no effect on bacterial burden and TOLL pathway activation in tick cells. QRT-PCR analysis showing bacterial burden (a) and *jak* (b), *stat* (c), *pgrp* (d), *tak1* (e), *toll* (f), *myd88* (g) or *pelle* (h) expression in control or EL-2 antibody-treated *A. phagocytophilum*-infected tick cells. Control and EL-2 antibodies were used at a concentration of 5µg/ml. *A. phagocytophilum* p44 levels were normalized to tick actin levels and mRNA levels of tick innate immune genes were normalized to tick 5.8S rRNA levels. *p*-value from student test is shown. Error bars indicate +/-standard error of the mean. Each circle represents data obtained from the sample collected from one well of the tick cells culture plate.

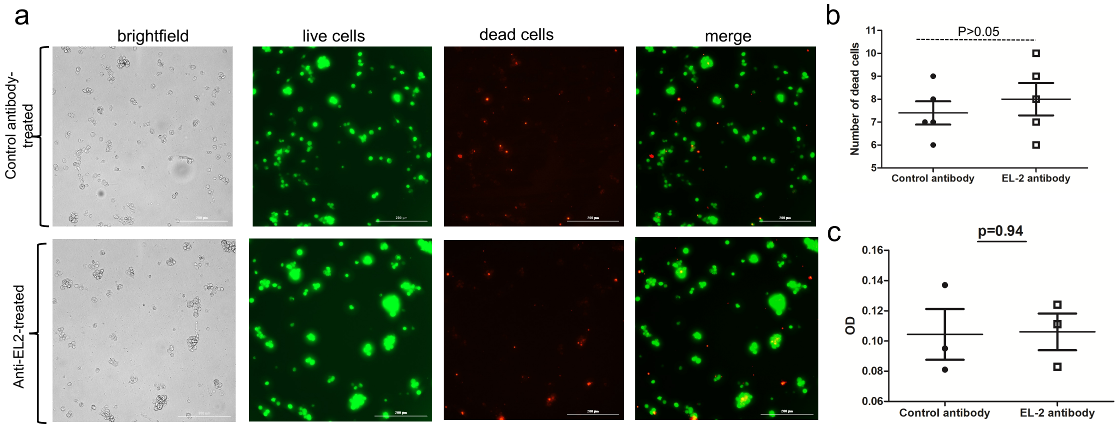

Supplementary Figure 3

Supplementary Figure 3. EL-2 antibody treatment has no cytotoxic effects on tick cells at 5µg/ml concentration. a) Phase contrast or fluorescent microscopic images showing live (green) or dead (red) uninfected tick cells. Uninfected tick cells were treated with either control or EL-2 antibodies at 5µg/ml concentration. After 24 h post treatment, cells were processed for Live/Dead staining assay, followed by imaging using fluorescent microscope. Scale bar indicates 200µm. b) Quantification of dead cells (red) from 5 images is shown. c) MTT assay showing viability of tick cells upon treatment with control or EL-2 antibodies at 5µg/ml concentration. Error bars indicate +/-standard error of the mean. P-value from student test is shown.

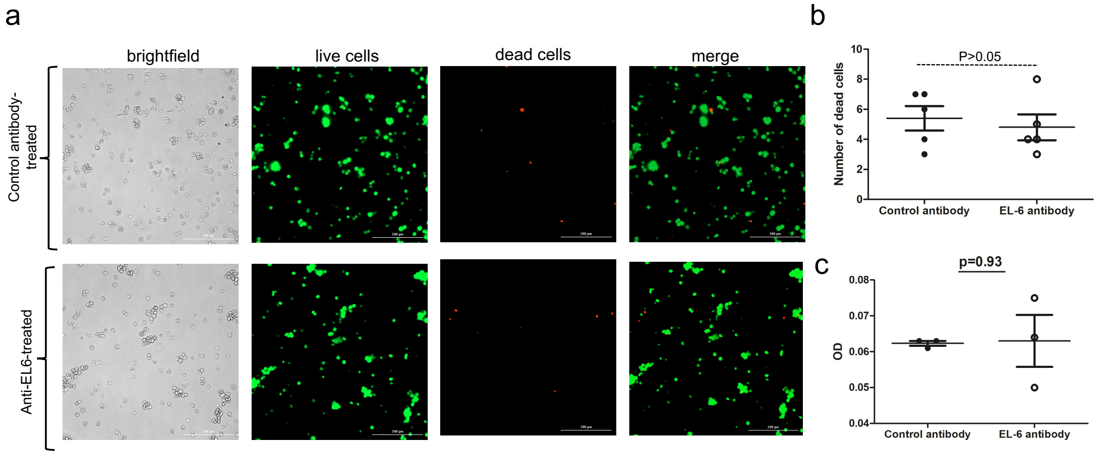

Supplementary Figure 4

Supplementary Figure 4. EL-6 antibody treatment has no cytotoxic effects on tick cells at 5µg/ml concentration. a) Phase contrast or fluorescent microscopic images showing live (green) or dead (red) uninfected tick cells. Uninfected tick cells were treated with either control or EL-6 antibodies at 5µg/ml concentration. After 24 h post treatment, cells were processed for Live/Dead staining assay, followed by imaging using fluorescent microscope. Scale bar indicates 200µm. b) Quantification of dead cells (red) from 5 images is shown. c) MTT assay showing viability of tick cells upon treatment with control or EL-6 antibodies at 5µg/ml concentration. Error bars indicate +/-standard error of the mean. P-value from student test is shown.

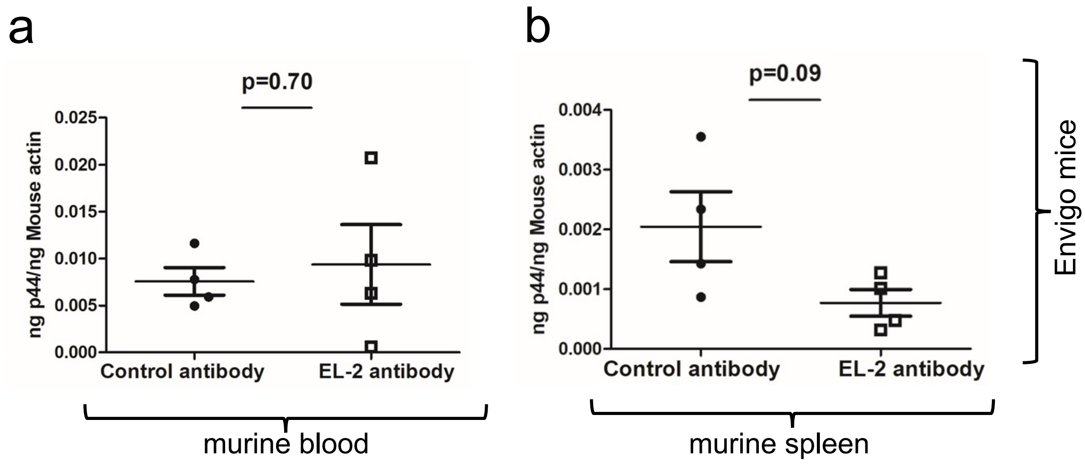

Supplementary Figure 5

Supplementary Figure 5. Passive immunization of mice with EL-2 antibody has no effect on tick-mediated transmission of *A. phagocytophilum*. QRT-PCR analysis showing *A. phagocytophilum* burden in control or EL-2 antibody-immunized murine blood (a) or spleen tissue (b). Immunizations were performed in mice obtained from Envigo. Bacterial loads were normalized to murine actin levels. Each circle represents data obtained from one mouse sample. Error bars indicate +/-standard error of the mean. P-value from student test is shown.

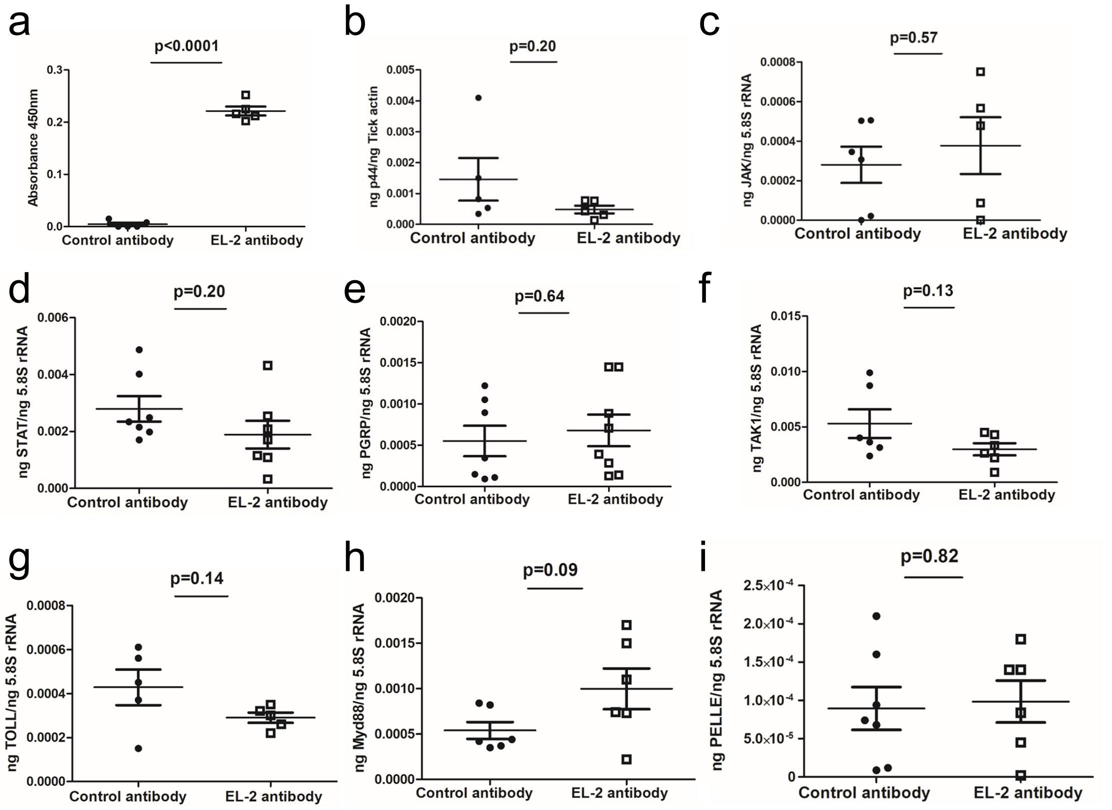

Supplementary Figure 6

Supplementary Figure 6. EL-2 antibody has no effect on bacterial loads and Toll pathway activation in engorged ticks. a) ELISA showing presence of EL-2 antibody in tick body. QRT-PCR analysis showing bacterial burden (b) and *jak* (c), *stat* (d), *pgrp* (e), *tak1* (f), *toll* (g), *myd88* (h) or *pelle* (i) expression in *A. phagocytophilum*-infected ticks fed on control or EL-2 antibody-immunized mice. Mice were immunized with control or EL-2 antibodies at a concentration of 15µg/mouse. *Anaplasma phagocytophilum p44* levels were normalized to tick actin levels and mRNA levels of tick innate immune genes were normalized to tick 5.8S rRNA levels. P-value from student test is shown. Error bars indicate +/-standard error of the mean. Each circle represents data obtained from the sample generated from one tick.

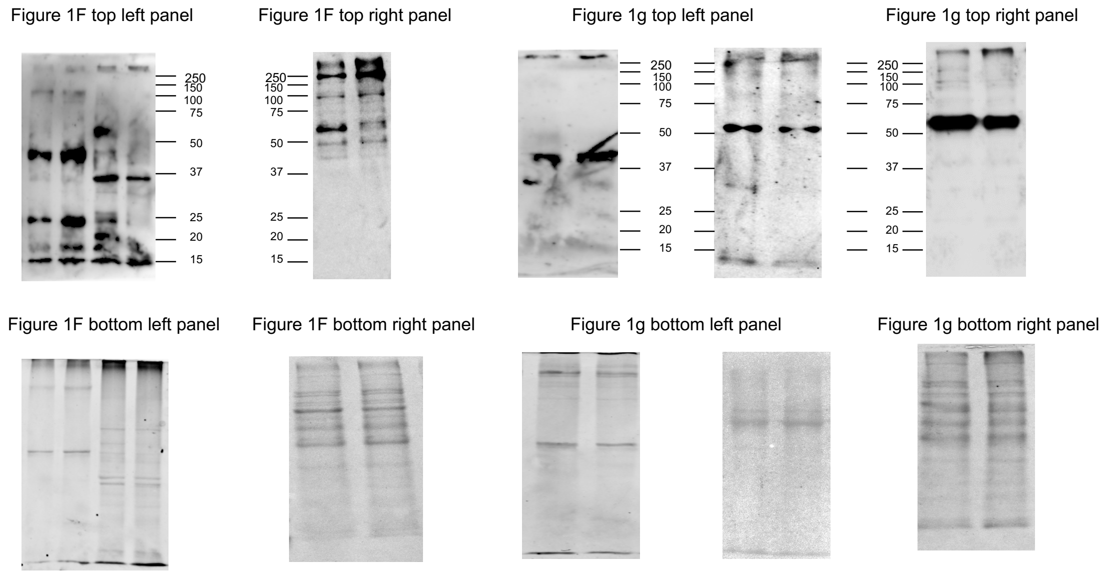

Supplementary Figure 7

Supplementary Figure 7. Full images of immunoblots, Coomassie staining and Ponceau staining that are shown in the main figure 1f and 1g are shown in this figure.
