# Supplementary material for: Immunization against arthropod protein impairs transmission of rickettsial pathogen from ticks to the vertebrate host
Source: NPJ Vaccines. 2023 May 30;8:79. doi: 10.1038/s41541-023-00678-y (PMC10229574; doi:10.1038/s41541-023-00678-y)
Supplement: Supplementary file 2 — REPORTING SUMMARY [file 41541_2023_678_MOESM2_ESM.pdf]

nature portfolio

Corresponding author(s): Girish Neelakanta

Last updated by author(s): Apr 20, 2023

Reporting Summary

Nature Portfolio wishes to improve the reproducibility of the work that we publish. This form provides structure for consistency and transparency in reporting. For further information on Nature Portfolio policies, see our Editorial Policies and the Editorial Policy Checklist.

Statistics

For all statistical analyses, confirm that the following items are present in the figure legend, table legend, main text, or Methods section.

n/a	Confirmed	
☐	☒	The exact sample size (*n*) for each experimental group/condition, given as a discrete number and unit of measurement
☐	☒	A statement on whether measurements were taken from distinct samples or whether the same sample was measured repeatedly
☐	☒	The statistical test(s) used AND whether they are one- or two-sided<br>*Only common tests should be described solely by name; describe more complex techniques in the Methods section.*
☒	☐	A description of all covariates tested
☒	☐	A description of any assumptions or corrections, such as tests of normality and adjustment for multiple comparisons
☐	☒	A full description of the statistical parameters including central tendency (e.g. means) or other basic estimates (e.g. regression coefficient) AND variation (e.g. standard deviation) or associated estimates of uncertainty (e.g. confidence intervals)
☒	☐	For null hypothesis testing, the test statistic (e.g. *F*, *t*, *r*) with confidence intervals, effect sizes, degrees of freedom and *P* value noted<br>*Give P values as exact values whenever suitable.*
☒	☐	For Bayesian analysis, information on the choice of priors and Markov chain Monte Carlo settings
☒	☐	For hierarchical and complex designs, identification of the appropriate level for tests and full reporting of outcomes
☒	☐	Estimates of effect sizes (e.g. Cohen's *d*, Pearson's *r*), indicating how they were calculated

Our web collection on statistics for biologists contains articles on many of the points above.

Software and code

Policy information about availability of computer code

Data collection	For quantification of dead cells, Images that were captured from Cytation 7 were used.
Data analysis	Data analysis was performed using GraphPad Prism 5 and Microsoft EXCEL Version 16.68 software.

For manuscripts utilizing custom algorithms or software that are central to the research but not yet described in published literature, software must be made available to editors and reviewers. We strongly encourage code deposition in a community repository (e.g. GitHub). See the Nature Portfolio guidelines for submitting code & software for further information.

Data

Policy information about availability of data

All manuscripts must include a data availability statement. This statement should provide the following information, where applicable:

- Accession codes, unique identifiers, or web links for publicly available datasets
- A description of any restrictions on data availability
- For clinical datasets or third party data, please ensure that the statement adheres to our policy

All data related to this manuscript are presented in the main figures or in supplementary figures. No data has been deposited in public databases.

Human research participants

Policy information about studies involving human research participants and Sex and Gender in Research.

Reporting on sex and gender	N/A, no Human subjects
Population characteristics	N/A, no Human subjects
Recruitment	N/A, no Human subjects
Ethics oversight	N/A, no Human subjects

Note that full information on the approval of the study protocol must also be provided in the manuscript.

Field-specific reporting

Please select the one below that is the best fit for your research. If you are not sure, read the appropriate sections before making your selection.

☒ Life sciences ☐ Behavioural & social sciences ☐ Ecological, evolutionary & environmental sciences

For a reference copy of the document with all sections, see nature.com/documents/nr-reporting-summary-flat.pdf

Life sciences study design

All studies must disclose on these points even when the disclosure is negative.

Sample size	Number of mice were chosen to obtain sufficient power between control or EL-2/EL-6 antibody treatment groups. For QRT-PCR, average of duplicates from independent samples were considered for data analysis.
Data exclusions	N/A
Replication	Minimum of 4 mice from two commercial vendors were considered in this study. All attempts at replication were successful.
Randomization	Mice were ordered at the same time from each vendor and randomly divided in to two-three groups (consisting of four mice per treatment).
Blinding	N/A. Mice were used for control-antibody or EL-6/EL-2 antibody treatment.

Reporting for specific materials, systems and methods

We require information from authors about some types of materials, experimental systems and methods used in many studies. Here, indicate whether each material, system or method listed is relevant to your study. If you are not sure if a list item applies to your research, read the appropriate section before selecting a response.

Materials & experimental systems

n/a	Involved in the study	
☐	☒	Antibodies
☐	☒	Eukaryotic cell lines
☒	☐	Palaeontology and archaeology
☐	☒	Animals and other organisms
☒	☐	Clinical data
☒	☐	Dual use research of concern

Methods

n/a	Involved in the study	
☒	☐	ChIP-seq
☒	☐	Flow cytometry
☒	☐	MRI-based neuroimaging

Antibodies

Antibodies used	Control, EL-2 and EL-6 antibodies were generated at GenScript, USA.
Validation	Antibodies were validated at the company and were analyzed by ELISA, Microscopy and Immunoblotting in the current study.

Eukaryotic cell lines

Policy information about cell lines and Sex and Gender in Research

Cell line source(s)	ISE6 tick cells and HaCaT cells, ATCC, USA.
Authentication	Cells were authenticated by ATCC and maintained as per the recommendation from ATCC.
Mycoplasma contamination	Cells were free of contamination.
Commonly misidentified lines (See ICLAC register)	N/A

Animals and other research organisms

Policy information about studies involving animals; ARRIVE guidelines recommended for reporting animal research, and Sex and Gender in Research

Laboratory animals	C3H/HeN (female mice, 4-6 weeks old, CharlesRiver Laboratories, USA) and C3H/HeNHsd (female mice, 4-6 weeks old, Envigo) and B6.129S7-Rag1tm1Mom/J mice (Jackson Laboratories, USA) were used in this study.
Wild animals	No Wild animals were used in this study.
Reporting on sex	All experiments were conducted with female mice.
Field-collected samples	No field-collected samples were used in this study.
Ethics oversight	All animal work in this study was performed based on the approved animal protocol from the University of Tennessee Institutional Animal Care and Use Committee (IACUC) with permit number 2801-0221.

Note that full information on the approval of the study protocol must also be provided in the manuscript.
